# Comparison of US Federal and Foundation Funding of Research for Sickle Cell Disease and Cystic Fibrosis and Factors Associated With Research Productivity

**DOI:** 10.1001/jamanetworkopen.2020.1737

**Published:** 2020-03-27

**Authors:** Faheem Farooq, Peter J. Mogayzel, Sophie Lanzkron, Carlton Haywood, John J. Strouse

**Affiliations:** 1 Deparment of Pediatrics and Medicine, Stony Brook University Hospital, Stony Brook, New York; 2 Division of Pediatric Pulmonology, Department of Pediatrics, Johns Hopkins University School of Medicine, Baltimore, Maryland; 3 Division of Hematology, Department of Medicine, Johns Hopkins University School of Medicine, Baltimore, Maryland; 4 Berman Institute of Bioethics, Johns Hopkins University School of Medicine, Baltimore, Maryland; 5 Division of Hematology, Department of Medicine, Duke University School of Medicine, Durham, North Carolina; 6 Division of Pediatric Hematology/Oncology, Department of Pediatrics, Duke University School of Medicine, Durham, North Carolina

## Abstract

**Importance:**

Sickle cell disease (SCD) and cystic fibrosis (CF) are severe autosomal recessive disorders associated with intermittent disease exacerbations that require hospitalizations, progressive chronic organ injury, and substantial premature mortality. Research funding is a limited resource and may contribute to health care disparities, especially for rare diseases that disproportionally affect economically disadvantaged groups.

**Objective:**

To compare disease-specific funding between SCD and CF and the association between funding and research productivity.

**Design, Setting, and Participants:**

This cross-sectional study examined federal and foundation funding, publications indexed in PubMed, clinical trials registered in ClinicalTrials.gov, and new drug approvals from January 1, 2008, to December 31, 2018, in an estimated US population of approximately 90 000 individuals with SCD and approximately 30 000 individuals with CF.

**Main Outcomes and Measures:**

Federal and foundation funding, publications indexed in PubMed, clinical trial registrations, and new drug approvals.

**Results:**

From 2008 through 2018, federal funding was greater per person with CF compared with SCD (mean [SD], $2807 [$175] vs $812 [$147]; *P* < .001). Foundation expenditures were greater for CF than for SCD (mean [SD], $7690 [$3974] vs $102 [$13.7]; *P* < .001). Significantly more research articles (mean [SD], 1594 [225] vs 926 [157]; *P* < .001) and US Food and Drug Administration drug approvals (4 vs 1) were found for CF compared with SCD, but the total number of clinical trials was similar (mean [SD], 27.3 [6.9] vs 23.8 [6.3]; *P* = .22).

**Conclusions and Relevance:**

The findings show that disparities in funding between SCD and CF may be associated with decreased research productivity and novel drug development for SCD. Increased federal and foundation funding is needed for SCD and other diseases that disproportionately affect economically disadvantaged groups to address health care disparities.

## Key Points

**Question:**

Are differences in disease-specific funding between sickle cell disease and cystic fibrosis associated with variations in drug development and research publications?

**Findings:**

This cross-sectional study of research funding and outputs for cystic fibrosis and sickle cell disease found that both federal funding and foundation expenditures were greater for cystic fibrosis compared with sickle cell disease. Significantly more research articles and drug approvals were found for cystic fibrosis compared with sickle cell disease, but the total numbers of clinical trials were similar.

**Meaning:**

The findings show that disparities in funding exist between sickle cell disease and cystic fibrosis and that these disparities may be associated with decreased research productivity and novel drug development for sickle cell disease.

## Teaser

This cross-sectional study compares disease-specific federal and foundation funding for sickle cell disease and cystic fibrosis research and assesses the factors associated with research productivity.

## Introduction

Sickle cell disease (SCD) and cystic fibrosis (CF) are inherited disorders associated with intermittent disease exacerbations that require hospitalizations and with a substantial reduction in the median life span. The US birth rate of SCD is 1 in 365 black individuals, and the US birth rate of CF is 1 in 2500 white individuals.^1,2,3^ Initially described in 1910, SCD subsequently became the first disease with a known molecular and genetic mechanism, making it the most thoroughly understood disease of its time.^4,5,6,7^ The initial knowledge of the molecular mechanism of SCD has not effectively translated into many approved therapies, but it has informed newborn screening and supportive care.^8,9^ In comparison, to our knowledge, CF was first described in the western literature in 1938.^10^ The genetic variant leading to a cellular membrane protein malfunction was not identified until 1989.^11,12,13^ The strategies used to identify the *CFTR* (OMIM 602421) variant informed techniques for the Human Genome Project and led to the approval of the first targeted therapy to correct the underlying chloride transport variant in 2011.^14,15,16^

National Institutes of Health (NIH) funding generally aligns with disease burden based on disease prevalence, severity, and age at onset. Several publications^17,18^ have examined the association between disability-adjusted life-years in the US and globally and NIH funding. These studies^17,18^ reported an association between increased funding for diseases that cause a greater reduction in disability-adjusted life-years. However, complex societal factors contribute to how private medical foundations generate revenue to fund advocacy and research. We assessed whether expenditures by the NIH and the national foundations for these diseases are associated with the number of publications indexed in PubMed, active clinical trials, and US Food and Drug Administration (FDA) drug approvals.

## Methods

For this cross-sectional study, we analyzed publicly reported metrics of disease funding and indicators of research productivity. This study was deemed to be exempt from institutional review board review based on criteria of the Health and Human Services Common Rule (45 CFR §46) because it did not include data from human participants, and informed consent was not required. This study followed the Strengthening the Reporting of Observational Studies in Epidemiology (STROBE) reporting guideline.

We report total NIH funding and career development awards for each disease from January 1, 2008, to December 31, 2017, using the NIH Report database. For funding from foundations, we reviewed publicly available Internal Revenue Service Form T-990 tax returns from disease-specific organizations. For SCD, we included 11 nonprofit organizations with at least 1 year of expenditures exceeding $500 000 during 2008 to 2017. Of the 110 Form T-990s, 9 were not available. For the missing forms, we imputed the mean expenses during the study period. We analyzed the Form T-990s for the 2 major CF organizations: the Cystic Fibrosis Foundation and Cystic Fibrosis Therapeutics (detailed expenditures in the eTable in the Supplement).

We developed a comprehensive search strategy with a medical informaticist to identify publications as a measure of research productivity. We performed a PubMed search from January 1, 1940, to December 31, 2018, which provided an overview of the research output of the 2 diseases over time. Two independent reviewers (including one of us [J.J.S.]) audited the search strategy and evaluated the results for validity.

We reviewed disease-specific US-based interventional trials on ClinicalTrials.gov from 2008 to 2018 using the search terms *sickle cell* and *cystic fibrosis*. Two independent reviewers (including one of us [J.J.S.]) audited the search results for validity. In addition, we divided the trials listed by funding source: (1) NIH and federal, (2) industry, and (3) other (foundation or university). We also reviewed the number of unique disease-specific FDA drug approvals and specific drug indications for each disease.

### Statistical Analysis

We compared values between SCD and CF using an unpaired *t* test. Statistical significance was set at a 2-tailed *P* < .05. Statistical analysis was performed using Excel (Microsoft).

## Results

Published estimates^19,20,21,22^ of approximately 90 000 individuals with SCD and approximately 30 000 individuals with CF from 2008 to 2018 were used (Table 1). The NIH funding per person with CF was greater than that for SCD (mean [SD], $2807 [$175] vs $812 [$147]; *P* < .001) (Table 2). The numbers of NIH career development awards for both diseases were similar (mean [SD], 16.6 [1.74] vs 16.7 [2.87]; *P* = .92) (Table 1). Philanthropic expenditures were significantly greater per person with CF compared with SCD (mean [SD], $7690 [$3974] vs $102 [$13.7]; *P* < .001).

**Table 1.** Summary of Disease Characteristics, Funding, and Research Output

	SCD	CF	*P* value
**Disease characteristics**			
Patients, No.^19,20,21,22^	90 000	30 000	NA
US birth incidence			
White	1/123 000	1/2600	NA
Black	1/314	1/6000	NA
Hispanic^1,2,3^	1/16 300	1/9200	NA
Life span, mean, y^20,23^	58	46	NA
US mortality in 2015, No.^24^	903	540	NA
Estimated lifetime costs per individual, $^25,26^	460 151	306 332	NA
**Annual funding (2008-2017)**			
NIH funding (in millions), mean (SD), $	76.3 (13)	84.2 (5.2)	.05
NIH funding per person affected, mean (SD), $	812 (147)	2807 (175)	<.001
Foundation expenditure (in millions), mean (SD), $	9.14 (1.2)	231 (119)	<.001
Foundation expenditure per person affected, mean (SD), $	102 (13.7)	7690 (3974)	<.001
Total funding per person affected, mean (SD), $	943 (148)	10 592 (3841)	<.001
Annual NIH career awards, mean (SD), No.	16.7 (2.87)	16.6 (1.74)	.92
**Research output (2008-2018)**			
Annual PubMed publications, mean (SD), No.	926 (157)	1594 (225)	<.001
Annual clinical trials, mean (SD), No.	24 (6.3)	27 (6.9)	.23
New FDA drug approvals, No.	1	4	NA
Novel FDA drug indications, No.	2	11	NA

Abbreviations: CF, cystic fibrosis; FDA, US Food and Drug Administration; NA, not applicable; NIH, National Institutes of Health; SCD, sickle cell disease.

**Table 2.** Disease-Specific NIH Funding and Combined Foundation Expenditures

Funding or expenditure	Year										Mean (SD)	*P* value
	2008	2009	2010	2011	2012	2013	2014	2015	2016	2017		
NIH funding (in millions), $												
SCD	80	63	73	65	65	70	75	75	92	105	76.3 (13.2)	.05
CF	90	86	86	79	86	78	77	80	89	91	84.2 (5.3)	
Per person affected												
SCD	889	700	811	722	722	778	833	833	1022	1167	812 (147)	<.001
CF	3000	2867	2867	2633	2867	2600	2567	2667	2967	3033	2807 (175)	
CF:SCD ratio of NIH funding per person	3.38	4.1	3.53	3.65	3.97	3.34	3.08	3.2	2.9	2.6	3.37 (0.46)	NA
Foundation expenditures (in millions), $												
SCD	10.3	9.83	9.27	8.42	7.38	7.73	8.03	9.13	11.2	10	9.14 (1.23)	<.001
CF	199	175	109	175	148	163	171	313	487	367	231 (119)	
Per person affected												
SCD	115	109	103	94	82	86	89	101	124	112	102 (13.7)	<.001
CF	6634	5823	3644	5816	4928	5443	5715	10 428	16 227	12 240	7690 (3974)	
CF:SCD ratio of foundation expenditures per person	58	53	35	62	60	63	64	103	131	109	75 (30)	NA

Abbreviations: CF, cystic fibrosis; NA, not applicable; NIH, National Institutes of Health; SCD, sickle cell disease.

**Figure 1.**
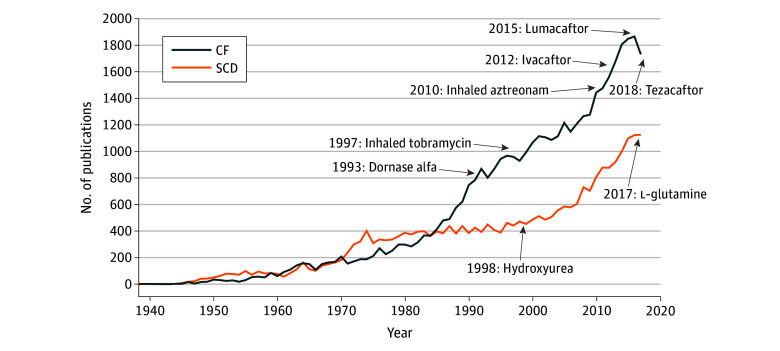
Number of Disease-Specific PubMed Listings and US Food and Drug Administration Drug Approvals Over Time CF indicates cystic fibrosis; SCD, sickle cell disease.

The number of PubMed publications per year was initially similar for the 2 diseases, but CF research output increased at a significantly quicker rate. During 2008 to 2018, annual CF publications remained greater than those of SCD (mean [SD] publications, 1594 [225] vs 926 [157]; *P* < .001) (Figure 1). Total interventional clinical trial listings on ClinicalTrials.gov for CF were greater than those for SCD from 2008 to 2018 (mean [SD] listings, 27.3 [6.9] vs 23.8 [6.3]; *P* = .22), but the difference was not statistically different. The SCD trials were more likely to be funded by NIH and federal funding (mean [SD], 5 [2.6] vs 1.9 [1.1]; *P* = .001) or foundation and university funding (mean [SD], 12.5 [6.1] vs 10.2 [2.9]; *P* = .27), but the difference for the latter was not statistically significant. The CF trials were significantly more likely to receive industry funding (mean [SD] trials, 15.6 [5.3] vs 6.8 [1.8]; *P* = .001) (Table 3).

**Table 3.** Annual Number of US-Based Interventional Clinical Trials Listed on ClinicalTrials.gov by Funding Source

Funding source	Year											Total	Mean (SD)	*P* value
	2008	2009	2010	2011	2012	2013	2014	2015	2016	2017	2018			
All^a^														
SCD	14	20	16	22	20	24	35	30	26	25	30	262	23.8 (6.3)	.22
CF	36	22	29	15	26	24	23	29	29	27	41	301	27.3 (6.9)	
NIH or other federal funding														
SCD	3	9	3	5	7	3	7	2	5	9	2	55	5 (2.6)	.001
CF	3	3	2	2	1	1	2	0	2	1	4	21	1.9 (1.1)	
Industry funding														
SCD	5	8	8	6	7	4	9	10	6	5	7	75	6.8 (1.8)	.001
CF	22	13	19	3	15	13	14	19	19	14	21	172	15.6 (5.3)	
Foundation or university funding														
SCD	6	4	6	11	7	18	19	18	16	12	21	138	12.5 (6.1)	.27
CF	12	7	8	10	11	10	7	10	8	12	17	112	10.2 (29)	

Abbreviations: CF, cystic fibrosis; NIH, National Institutes of Health; SCD, sickle cell disease.

^a^ Three trials for CF and 5 trials for SCD had dual funding sources.

Disease-specific drug development also favored CF (4 vs 1 drug approvals) (Table 1). There were 6 disease-specific drugs for CF compared with 2 for SCD (Figure 1). Since 2012, the 3 novel disease-specific drugs that were approved for CF received 5 new indications. For SCD, only hydroxyurea received a new indication in 2017, and l-glutamine was initially approved for SCD the same year.

## Discussion

Despite SCD being 3 times as prevalent as CF, both diseases received a similar amount of federal government research funding between 2008 and 2018. The funding disparity was markedly increased when factoring in disease-specific private foundation funding. The additional research support was associated with greater research productivity and pharmaceutical development for CF compared with SCD.

### Federal Disease-Specific Funding and Disease Burden

The NIH allocates research funds in accordance with disease burden.^18^ Heart disease and cancer receive the largest amounts of funding because they are associated with significant morbidity and mortality for millions of people. Although SCD is 3 times as prevalent as CF, both diseases receive approximately equal NIH funding. It is challenging to calculate methods of disease burden, such as disability-adjusted life-years, for chronic genetic diseases. However, CF and SCD are associated with substantial health care–related costs, especially for hospital care, and have considerable effects on daily life.^25,26,27^ With the exception of recently developed *CFTR* modulator therapies, approximately 80% of the health care cost associated with each disease is spent on hospital care.^25,27^ The cost of health care for patients increases with age; thus, as more patients grow into adulthood, costs are expected to increase further.^25^

### Philanthropic Foundation Funding

**Figure 2.**
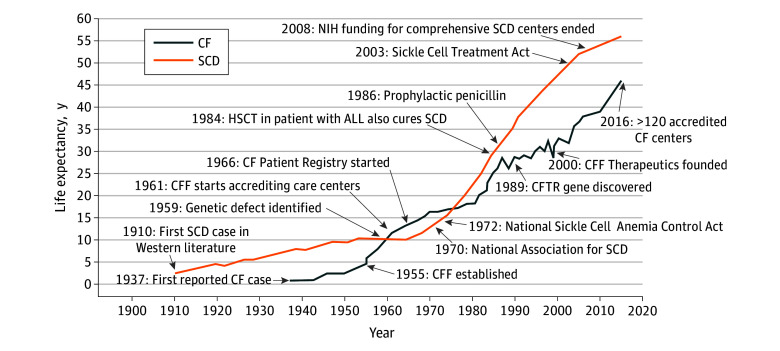
Progression of Life Span for Cystic Fibrosis (CF) and Sickle Cell Disease (SCD) and Major Health Care Milestones Life Span graphs adapted from Wailoo^29^ and Elborn.^30^ ALL indicates acute lymphocytic leukemia; CFF, Cystic Fibrosis Foundation; HSCT, hematopoietic stem cell transplant; and NIH, National Institutes of Health.

The size and revenue of medical charities generally reflect the disease burden. The American Heart Association and American Cancer Society are the 2 largest nonprofit health care organizations in the United States.^28^ However, when a disease relies heavily on foundation expenditures, disparities in funding can have a substantial effect on research productivity and clinical care. Our study found disparities in foundation funding between SCD and CF. The funding discrepancy was, to our knowledge, first reported in 1970 by Robert Scott,^8,9^ who framed SCD as a neglected public health crisis. The articles by Scott, along with the establishment of the National Association for Sickle Cell Disease in 1970, contributed to the passage of the National Sickle Cell Anemia Control Act in 1972 (Figure 2).^29,30,31^ This initiative increased federal funding and community-based screening for the disease. However, despite this public-private effort, NIH and foundation funding for SCD has remained low compared with CF.^32^ Fundraising capacity for private charitable organizations relies heavily on advocacy and donors who have the capacity to contribute. Even though sources of charitable funding are diverse, most of the SCD community is black.^1^ Despite black individuals donating a substantial portion of their income for philanthropy, there are many competing societal demands for charitable donations.^28^ In addition, there is historical distrust of the medical establishment among the black community, contributing to decreased funding and participation in medical research.^33^ The community affected by SCD may benefit from increased awareness and media exposure to increase advocacy to support federal and private investment in research.

### Research Productivity

Measuring the association between research funding and productivity is complex and controversial. Although funding and grants can be easily measured, quantifying research productivity is challenging.^34^ Our data revealed that CF funding and research publications were consistently greater than those for SCD. The magnitude of funding disparity was substantially greater than the magnitude of measurable research disparity. Research publications were similar (Figure 1).

### Pharmaceutical Funding and Drug Development

Recent breakthrough research and discoveries have been in biopharmaceuticals. Despite the slowing rate of federal government pharmaceutical funding, pharmaceutical funding from the private sector has been increasing, with an estimated $90 billion spent annually in research and development.^35,36^ In addition, there has been a renewed focus on drug development for rare diseases that affect fewer than 200 000 people. Since 2013, 60% of breakthrough therapies that have obtained FDA approval were designated as orphan drugs under the 1983 Orphan Drug Act.^37^

Both CF and SCD are classified as rare diseases, and new products are eligible for orphan drug incentives; however, it is challenging to ascertain disease-specific investment by the pharmaceutical industry. Data from ClinicalTrials.gov showed that there have been more than twice as many industry-funded trials for CF compared with SCD. In addition, the Cystic Fibrosis Foundation’s venture philanthropy model has helped produce breakthrough therapies for CF.^14,15,16^ The unified Cystic Fibrosis Foundation allows for the investment of large sums in early-stage drug development, which has contributed to novel *CFTR* modulator therapies. The present analysis did not directly include payment in 2014 of $3.3 billion to the Cystic Fibrosis Foundation for the royalties from ivacaftor. This large lump sum has been reinvested for further research, drug development, and quality improvement efforts for persons with CF.^38^ Although this is an atypical 1-time occurrence, the magnitude of the transaction may have far-reaching effects and may be associated with increased disparity in private funding between these 2 diseases. The success of this venture philanthropy model can be seen in the increased Cystic Fibrosis Foundation funding beginning in 2015 and can support an additional $158 million dollars of annual expenditures at a spending rate of 4.8% (mean rate for endowment over $1 billion from 2000 to 2016).^39^

In contrast, the few breakthroughs in SCD management occurred almost incidentally. In 1984, a patient with acute lymphoid leukemia and SCD underwent hematopoietic stem cell transplant, which also cured his SCD.^40^ The same year, hydroxyurea, once a chemotherapy agent, was found to increase fetal hemoglobin levels in SCD.^41^ However, hydroxyurea was not approved by the FDA until 1998 for the treatment of adults with severe SCD. A specific FDA indication for children was not obtained until 2017. The development of targeted therapies for CF within 25 years of discovering the genetic mechanism of the disease further accentuates the almost century-long drought in drug development for SCD.^42^ However, there were multiple advancements in SCD therapies in 2017. L-glutamine was the first disease-specific therapy developed for SCD to gain FDA approval.^43^ The same year, crizanlizumab, a humanized monoclonal antibody, demonstrated efficacy in reducing vasoocclusive crises, and the first case report of a patient undergoing successful lentiviral vector–mediated gene therapy for SCD was published.^44,45^

Despite substantial advancements, CF and SCD therapies have notable shortcomings. Targeted therapies are effective for only a certain percentage of patients with CF and are not curative agents.^46^ Therapies have improved markers of disease activity, such as forced expiratory volume and reduced pulmonary exacerbations.^14,15,16^ Analogously, therapies for SCD have also improved fetal hemoglobin levels and reduced vasoocclusive crises.^43,45,47,48^ Although hematopoietic stem cell transplant is curative for SCD, the risk of treatment-related mortality, late adverse effects, and lack of eligible donors have limited uptake of this therapy.^49,50,51^ Going forward, CF and SCD may benefit from the renewed focus on the development of orphan drugs, precision medicine, and gene therapy^52^

### Access to Quality Comprehensive Care

Novel disease-modifying therapies may be associated with improved survival for the population with CF, but the standard of the successful CF care model is comprehensive, multidisciplinary care obtained in specialized care centers. Multidisciplinary disease-specific comprehensive care centers have emerged to care for people with genetic diseases.^53^ The influence of the Cystic Fibrosis Foundation has involved research funding and the implementation of national quality-of-care standards the accredited comprehensive centers must uphold. There are more than 120 comprehensive care centers for CF in total, and 100 centers also provide adult care. In comparison, federal funding for 10 sickle cell centers ended in 2008; thus, there are no longer federally supported centers for comprehensive SCD care.^53^ Existing SCD comprehensive centers typically depend on institutional support because third-party reimbursement for clinical services is generally low; most individuals with SCD have Medicaid coverage, which is associated with decreased access to high-quality care and more emergency department use.^54^

National patient registries for CF are used to evaluate adherence to guidelines, benchmark CF centers, and provide data for quality improvement efforts.^55^ For SCD, evidence-based guidelines exist, but the adaptation of best practices has been variable.^56^ For example, a previous study^57^ suggested that only 25% of eligible adults with SCD are prescribed hydroxyurea despite the drug’s proven efficacy since the early 1990s. The substantial improvement in life expectancy in patients with SCD and CF may be associated with the earlier and optimal application of supportive care and disease-modifying therapies. Most patients with CF and SCD in the United States now reach adulthood.^58,59^ Although childhood mortality related to SCD has steadily improved, the number of deaths from SCD among adults has been increasing.^60^

Despite the differences in funding, the overall life expectancy of patients with SCD has increased at a faster rate than that of patients with CF during the past 2 decades (Figure 2). This finding may reflect greater disease severity of CF or a less representative sample for SCD because most recent survival studies^23,61^ for SCD have been limited to adults followed up at a single center of excellence. For example, a previous analysis^62^ demonstrated that the increased life expectancy of patients with CF observed in Canada compared with the US may be associated with insurance status in the US and access to lung transplants. This finding underscores the need for the communities involved with CF and SCD to develop an infrastructure to ensure access to optimal care for affected individuals.

### Race/Ethnicity and Stigma

The role of race/ethnicity in the context of health care disparities in the US is well documented.^63^ Consideration of SCD as a black disease in the US has permeated the experience for patients since the first description in the Western medical literature.^64^ Even initial screening efforts for SCD were partially motivated by racial/ethnic undertones.^65^ CF has been recognized as a predominantly white disease; however, health care disparities associated with race/ethnicity also affect Hispanic individuals with CF. There is increased mortality among Hispanic patients with CF and relative underrepresentation in clinical trials.^66,67^ The interaction of black individuals with the health care system is associated with distrust given past ethical violations in the name of medical progress.^68^ This distrust between patient and practitioner can lead to conflict that results in suboptimal medical care and worsens patient medication adherence.^69^

Stigma for people with SCD is most apparent when they are in the emergency department with severe pain and require compassionate, evidence-based care.^70^ In comparison, a measured decrease in lung function that requires airway clearance and antibiotics during a CF pulmonary exacerbation is not as stigmatizing as the report of severe pain that requires opiates during a vasoocclusive crisis. The recommended treatment for acute sickle cell pain involves medications associated with abuse, misuse, and addiction, which further challenges the clinical decision-making of practitioners. Not only are individuals with SCD already stigmatized as drug seeking, the nationwide focus on the opioid epidemic poses a new challenge for individuals in pain. Increasing disease awareness, educating practitioners, and developing coordinated care models can help mitigate stigma.^71^

### Recommendations

A robust national organization linked with state and local chapters can pool funds to increase research funding, clinical trials, novel therapeutics, and develop interconnected comprehensive care centers. This approach has been successfully modeled by the Cystic Fibrosis Foundation,^72^ and patients with SCD may benefit from similar approaches. Current charitable SCD organizations are disjointed and have limited success with fundraising given reliance on small donations from the community affected by the disease. Robust financial support from established large foundations appears to be necessary to fund advocacy efforts and breakthrough research projects. Effective advocacy involves leveraging the changing media landscape to generate disease interest and develop corporate and community partnerships to boost funding.^73^

We believe that the federal government should increase funding for SCD given the gap in private support and the association of funding with quality of life and survival. Federal legislative advocacy should also involve reestablishing federally funded comprehensive SCD treatment centers to complement existing support from the Health Resources and Services Administration for SCD Treatment Demonstration Regional Collaboratives.^74^ Research, education, and clinical care are shared missions of academic centers, and there appears to be opportunity for improvement in all 3 domains with regard to SCD. The more complex societal challenge involves overcoming mistrust and racism to empower and engage a community affected by the disease that has been historically disenfranchised.^75^ In addition, we propose partnerships among SCD practitioners, patient advocates, public health officials, and third-party payers to invest in improved comprehensive care for persons with SCD.

### Limitations

This study has limitations. We cannot account for research funding by the NIH or other foundations that are not specific to SCD or CF. It is challenging to ascertain disease-specific investment by the pharmaceutical industry. This analysis also did not directly include the 1-time lump sum of $3.3 billion generated by the Cystic Fibrosis Foundation from royalties from the sale of ivacaftor.^38^ The shortcomings in using disease-specific bibliometrics, such as number of publications and citations, are that they generally do not capture the true value of breakthrough accomplishments and discoveries.

## Conclusions

The findings show that disparities in funding between SCD and CF may be associated with decreased research productivity and novel drug development for SCD. Increased federal and foundation funding is needed for SCD and other diseases that disproportionately affect economically disadvantaged groups to address health care disparities.

## Notes

Supplement. **eTable.** Annual Expenditures for Private Foundations
