# Supplementary material for: Comparison of US Federal and Foundation Funding of Research for Sickle Cell Disease and Cystic Fibrosis and Factors Associated With Research Productivity
Source: JAMA Netw Open. 2020 Mar 27;3(3):e201737. doi: 10.1001/jamanetworkopen.2020.1737 (PMC12549141; doi:10.1001/jamanetworkopen.2020.1737)
Supplement: Supplement. — eTable. Annual Expenditures for Private Foundations [file jamanetwopen-e201737-s001.pdf]

# Supplementary Online Content

Farooq F, Mogayzel PJ, Lanzkron S, Haywood C, Strouse JJ. Comparison of US federal and foundation funding of research for sickle cell disease and cystic fibrosis and factors associated with research productivity. *JAMA Netw Open*. 2020;3(3):e201737. doi:10.1001/jamanetworkopen.2020.1737

**eTable.** Annual Expenditures for Private Foundations

This supplementary material has been provided by the authors to give readers additional information about their work.

© 2020 Farooq F et al. *JAMA Network Open.*

| **Cystic Fibrosis Foundations** | | | | | | | | | | | |
|---|---|---|---|---|---|---|---|---|---|---|---|
| | 2008 | 2009 | 2010 | 2011 | 2012 | 2013 | 2014 | 2015 | 2016 | 2017 | Annual Average |
| CFF | 139419792 | 121319145 | 67973496 | 125721614 | 79288914 | 75047721 | 80021438 | 192986761 | 299650531 | 292766690 | 147419610.2 |
| CFT | 59607050 | 53381907 | 41334760 | 48755900 | 68537526 | 88243426 | 91438241 | 119857486 | 187173457 | 74437028 | 83276678.1 |
| SUM | 199026842 | 174701052 | 109308256 | 174477514 | 147826440 | 163291147 | 171459679 | 312844247 | 486823988 | 367203718 | 230696288.3 |
| Per Person | 6634 | 5823 | 3644 | 5816 | 4928 | 5443 | 5715 | 10428 | 16227 | 12240 | 7690 |
| **Sickle Cell Foundations** | | | | | | | | | | | |
| | 2008 | 2009 | 2010 | 2011 | 2012 | 2013 | 2014 | 2015 | 2016 | 2017 | Annual Average |
| SCDAA | 1670951 | 1806565 | 1399062 | 1185023 | 783974 | 824760 | 951094 | 1902300 | 3805147 | 2727128 | 1705600 |
| James Clark | 942311 | 833422 | 869801 | 829001 | 904694 | 990041 | 983813 | 916596 | 940090 | 917464 | 912723 |
| SCF Palm Beach | 1341465 | 1246642 | 1279007 | 1162574 | 784171 | 769578 | 814078 | 883916 | 806206 | 1009737 | 1009737 |
| SCD CA | 718175 | 634082 | 603329 | 330819 | 248617 | 272164 | 275996 | 548770 | 444876 | 452981 | 452981 |
| SCD FL | 644281 | 730251 | 568742 | 443060 | 374565 | 420426 | 790133 | 390151 | 386947 | 527617 | 527617 |
| SCD GA | 1300732 | 1075647 | 940671 | 869329 | 844752 | 792822 | 671198 | 841356 | 1019531 | 928449 | 928449 |
| SCD IL | 548271 | 606962 | 432998 | 418345 | 565009 | 601850 | 552880 | 55763 | 427784 | 328723 | 453859 |
| SCD Phil | 559618 | 578672 | 578830 | 390192 | 342159 | 307432 | 375199 | 401290 | 361846 | 432804 | 432804 |
| SCD AL | 706833 | 727923 | 930807 | 947532 | 685392 | 976919 | 740354 | 946295 | 832757 | 832757 | 832757 |
| SCD MICH | 1447675 | 1170255 | 1238821 | 1410003 | 1460351 | 1337159 | 1508781 | 1786869 | 1669162 | 1447675 | 1447675 |
| Martin Center | 474681 | 422882 | 423150 | 431250 | 386788 | 438020 | 370161 | 455999 | 486464 | 432155 | 432155 |
| SUM | 10354993 | 9833303 | 9265218 | 8417128 | 7380472 | 7731171 | 8033687 | 9129305 | 11180810 | 10037490 | 9136358 |
| Per Person | 115 | 109 | 103 | 94 | 82 | 86 | 89 | 101 | 124 | 112 | 102 |

eTable. Annual Expenditures for Private Foundations
All values reported in dollar amounts.
